# Carotenoids as a Protection Mechanism against Oxidative Stress in *Haloferax mediterranei*

**DOI:** 10.3390/antiox9111060

**Published:** 2020-10-29

**Authors:** Micaela Giani, Rosa María Martínez-Espinosa

**Affiliations:** 1 Biochemistry and Molecular Biology Division, Agrochemistry and Biochemistry Department, Faculty of Sciences, University of Alicante, Ap. 99, E-03080 Alicante, Spain; micaela.giani@ua.es; 2 Multidisciplinary Institute for Environmental Studies “Ramón Margalef”, University of Alicante, Ap. 99, E-03080 Alicante, Spain

**Keywords:** archaea, bacterioruberin, haloarchaea, H_2_O_2_, oxidative stress, hydrogen peroxide

## Abstract

Haloarchaea are extremophilic microorganisms that in their natural ecosystem encounter several sources of oxidative stress. They have developed different strategies to cope with these harsh environmental conditions, among which bacterioruberin production is a very notable strategy. Bacterioruberin (BR) is a C_50_ carotenoid synthesized in response to different types of stress. Previous works demonstrated that it shows interesting antioxidant properties with potential applications in biotechnology. In this study, *Haloferax mediterranei* strain R-4 was exposed to different concentrations of the oxidant compound H_2_O_2_ to evaluate the effect on carotenoid production focusing the attention on the synthesis of bacterioruberin. *Hfx. mediterranei* was able to grow in the presence of H_2_O_2_ from 1 mM to 25 mM. Cells produced between 16% and 78% (w/v) more carotenoids under the induced oxidative stress compared to control cultures. HPLC-MS analysis detected BR as the major identified carotenoid and confirmed the gradual increase of BR content as higher concentrations of hydrogen peroxide were added to the medium. These results shed some light on the biological role of bacterioruberin in haloarchaea, provide interesting information about the increase of the cellular pigmentation under oxidative stress conditions and will allow the optimization of the production of this pigment at large scale using these microbes as biofactories.

## 1. Introduction

Oxidative stress arises from an imbalance between the production of reactive oxygen (ROS) and nitrogen species (NOS), and the antioxidant mechanisms of the living organism like enzymes (i.e., catalase) or other molecules such as glutathione, vitamins, or pigments [1,2]. The exposure to oxidants, such as superoxide anion (O^•−^), hydrogen peroxide (H_2_O_2_), and hydroxyl radical (HO^•^), usually causes damage to a wide spectrum of biomolecules and cellular structures such as cell membranes, nucleic acids, or proteins [3,4]. 

Haloarchaea are extremophilic microorganisms that inhabit hypersaline environments since they require mid or high salt concentrations to survive. Their natural ecosystems comprise hypersaline lakes, soils, springs, solar salterns, and rock salt deposits, where not only the level of salinity is harsh, but also other environmental parameters are extreme, such as intense solar radiation and frequent desiccation-hydration cycles [5,6]. Therefore, halophilic archaea are frequently exposed to oxidative stress and for this reason, they have developed different strategies to cope with these conditions. Among the adaptations, several DNA repair mechanisms [6], synthesis of polymers used as carbon and energy storage (PHA/PHB/PHV) [7,8,9], or hyperpigmentation [6,10,11,12,13,14] can be highlighted.

The saline soils or aquatic hypersaline environments usually inhabited by haloarchaea show a peculiar characteristic in summer due to their intense red and pink coloring, which is caused by the accumulation of carotenoids in the cell membranes of haloarchaeal populations as well as of halophilic bacteria such as *Salinibacter ruber* or eukaryotic organisms such as *Dunaliella salina* [15]. Haloarchaea synthesize mainly the rare type of carotenoid called bacterioruberin and its derivatives [12,16]. Bacterioruberin (BR) is characterized by a higher number of carbon units: 50 units (C_50_) instead of 40, as in β-carotene (C_40_) [11]. BR plays an important role protecting cells against oxidative damage, given its attributed strong antioxidant properties, which are related mainly to the higher number of conjugated double bonds and the length of the carbon chain. The structure of BR contains 13 pairs of conjugated double bonds in contrast with the nine pairs of β-carotene, which make this carotenoid a superior radical scavenger than C_40_ carotenoids [6,11].

Several factors are involved in the regulation of carotenoid biosynthesis in haloarchaea, as for example salinity [10], pH [13], oxygen, and light exposure [16,17]. Haloarchaea require the presence of light to utilize their proton pump, but in the process, they might come upon photodamage. Haloarchaea protect themselves from photodamage using the antioxidant activity of carotenoids by means of ROS scavenging [6,18]. Furthermore, it is worth mentioning that carotenoids remain intact after oxidative damage prevention [19].

Although haloarchaeal carotenoids show interesting properties, data about the carotenogenesis pathway in haloarchaea are limited and there is a lack of precision in the annotation of most genes involved. In 2015, three essential genes for the synthesis of BR were identified in *Haloarcula japonica*: *c0507*, *c0506*, and *c0505*. These genes encode three enzymes responsible of the conversion from lycopene to BR in *Haloarcula japonica*, which are carotenoid 3,4-desaturase (CrtD), a bifunctional lycopene elongase and 1,2-hydratase (LyeJ), and a C50 carotenoid 2,3-hydratase (CruF) [20]. More recently, a bioinformatic analysis has studied several haloarchaeal genomes to propose a potential carotenogenesis pathway in haloarchaea. A three-gene cluster was reported in all studied haloarchaeal species, encoding a phytoene desaturase, a prenyltransferase, and a currently unidentified carotenoid biosynthesis protein, which shows some similarity with the CruF identified in *Haloarcula japonica* [21].

H_2_O_2_ is a natural intracellular by-product of aerobic metabolism, being the most abundant ROS found in vivo. Nevertheless, its concentration must be kept under homeostatic control given its strong oxidant activity [22,23]. Nowadays, it is widely accepted that this low molecular weight molecule plays an important role in metabolic regulation comparable to the activity of diffusible gases such as NO, CO, or H_2_S [24].

There is scarce information about the in vitro response of haloarchaea against H_2_O_2_-induced oxidative stress. VNG0258H archaeal transcription factor was identified as a regulator of the adaptation to oxidative stress conditions induced by a range of concentrations from 5 to 25 mM H_2_O_2_ in *H. salinarum* [25]. Recently, hundreds of differentially expressed sRNAs have been identified in response to 2 mM H_2_O_2_-induced oxidative stress in *Hfx. volcanii* [26].

Although it is quite clear that BR is key molecule among the strategies to deal with oxidative stress, there is no experimental evidence regarding the tolerance of *Hfx. mediterranei* to different concentrations of an oxidizing agent such as H_2_O_2_. Moreover, the overall capability to synthesize carotenoids (mainly BR) when growing under oxidizing conditions is also unknown. To determine the impact of oxidative stress on the overall production of carotenoids, particularly BR, *Hfx. mediterranei* was grown under standard conditions previously described in the literature (complex medium) [16,27] and hydrogen peroxide-induced oxidative stress conditions. These results shed some light on the biological role of bacterioruberin in haloarchaea, provide interesting information about the increase of the cellular pigmentation under oxidative stress conditions, and will allow the optimization of the production of this pigment at large scale using these microbes as biofactories.

## 2. Materials and Methods

### 2.1. Culture Growth Conditions

*Hfx. mediterranei* strain R-4 (ATCC33500) (isolated from saltern ponds located in Santa Pola, Alicante, Spain) was used for all experiments. Cells were grown in a complex medium containing 25% (w/v) of inorganic salts [27] and 0.5% (w/v) yeast extract. The pH was adjusted to 7.3 using NaOH and HCl solutions. Growth conditions included 42 °C and shaking at 170 rpm (Infors HT Multitron Standard). Growth specific velocity (µ) (1) and duplication time (Dt) (2) were calculated for each condition using the following equations:

µ = ln (X − X_0_)/(t − t_0_) (1)

Dt = ln(2)/µ(2)

### 2.2. Oxidative Stress Exposure

*Hfx. mediterranei* strain R-4 was exposed to different concentrations of the oxidant H_2_O_2_ (1, 2.5, 5, 10, 12.5, 15, 17.5, 20, 22.5, 25, 30, 50, and 75 mM) in biological triplicates. Fifty mL cultures were grown in 50 mL Erlenmeyer under optimal conditions to an optical density (OD) of 0.8 (mid-exponential phase). With exception of the control (no treatment), H_2_O_2_ from a 33% (v/v) stock was directly added to the cultures to reach the final concentrations mentioned above. Then, they were incubated until stationary phase (when most bacterioruberin is synthesized) at 42 °C with shaking at 170 rpm. When cultures reached stationary phase, they were centrifuged at 7830× *g* for 30 min to remove the supernatant. The wet pellets were stored at −20 °C until their use for carotenoid extraction.

### 2.3. Carotenoid Extraction and BR Quantification

Cell pellets were suspended in pure acetone, incubated at 4 °C overnight under gentle continuous agitation and then centrifuged (7830 rpm, 30 min) to obtain the carotenoid extract. This step was repeated until cells were decolorated. BR optical density (OD) at 494 nm was determined in duplicates and its concentration was calculated using Equation (3) [16]. Acetone-BR extracts were stored at −20 °C for further analysis by UV-Vis and HPLC.

mg·L^−1^= (OD_494_/2540) × 10^4^ (3)

### 2.4. UV-VIS and HPLC Analysis

Absorption UV–visible spectra were obtained at room temperature on a Cary 60 UV-Vis spectrophotometer (Agilent, Santa Clara, CA, USA) using the scan mode, with a 300–800 nm absorbance range. Acetone was used as blank and baseline correction.

The HPLC analysis of carotenoids in acetone was performed using a Zorbax extended -C18 column (Agilent, Santa Clara, CA, USA) (2.1–50 mm, 1.8 m) on an Agilent 1200 series system (Santa Clara, CA, USA). The optimization and validation of HPLC parameters were determined in previous experiments [16]. To determine the mass spectra of the different compounds, a 6490 Triple Quad LC/MS system (Agilent, Santa Clara, CA, USA) was used equipped with an electrospray ionization source (ESI) Jet stream operating in positive scan mode (m/z range of 300–900), with 0.1 a.m.u. (atomic mass unit). precision, and controlled by MassHunter Workstation Software (Agilent, B.05.00, Santa Clara, CA, USA). The following specific working conditions were used: capillary voltage 3000 V, gas flow rate 11 L min^−1^, gas temperature 290 °C, sheath gas flow rate 12 L min^−1^, sheath gas temperature 300 °C and nebulizer pressure 35 psi.

### 2.5. Statistics

One-way ANOVA followed by Dunnett’s multiple comparisons test was performed using GraphPad Prism version 7.00 for Windows (GraphPad Software, CA, USA, www.graphpad.com). Data are presented as the average of three independent experiments in the case of growth curves and growth specific velocity determination; and biological duplicates in BR quantification; and BR and MABR relative percentage. Statistical significance was determined by *p*-value at *p* < 0.05.

## 3. Results

### 3.1. Hfx. medit Erranei Tolerance to H_2_O_2_

**Figure 1:**
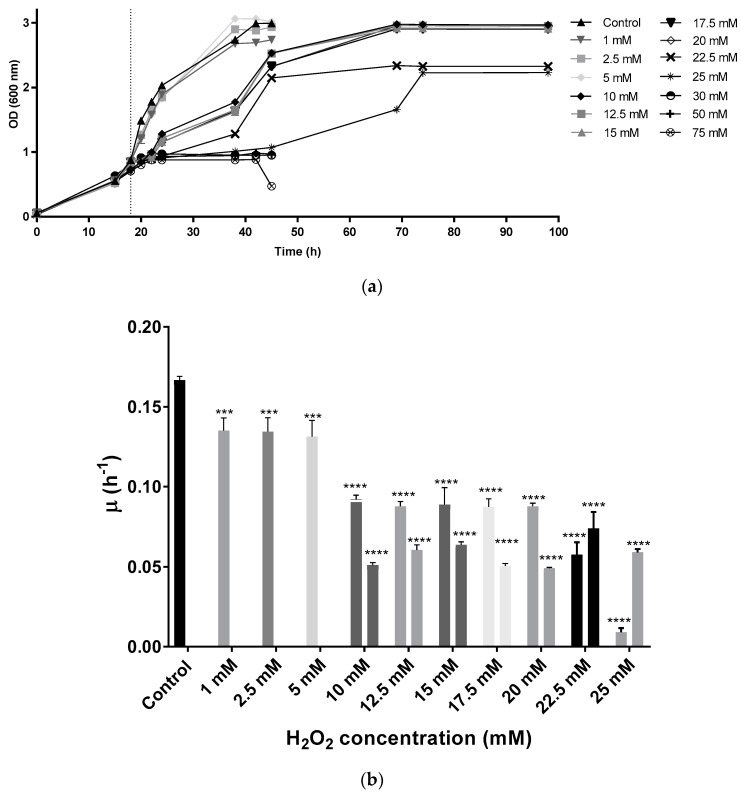
(**a**) Growth curve of *Hfx. Mediterranei* cell cultures. Vertical dotted line indicates the time of the H_2_O_2_ addition. (**b**) Specific growth velocity (µ) of *Hfx. Mediterranei* during exponential phase. From 10 mM to 25 mM, there are two µ values since under these conditions, cells presented a diauxic growth with two exponential phases. Each experimental sample was compared to the control to evaluate statistical significance. *** *p* < 0.001. **** *p* < 0.0001.

*Hfx. mediterranei* strain R-4 was grown in complex media without stress until the cultures reached an OD equal to 0.8 (mid-exponential phase of growth). Then, oxidative stress was induced by adding different concentrations of the oxidant H_2_O_2_ (1, 2.5, 5, 10, 12.5, 15, 17.5, 20, 22.5, 25, 30, 50, and 75 mM as final concentrations). These results demonstrate that *Hfx. mediterranei* could grow in the presence of up to 25 mM of H_2_O_2_. At the lowest concentrations (1, 2.5, and 5 mM) growth curves showed the typical behavior expected from standard complex media (lag phase is followed by exponential phase, and finally, a stationary phase is observed). From 10 mM to 25 mM, cells showed diauxic growth with two clear growth phases. When exposed to 30 mM or higher, cells reached the stationary phase at an optical density around 1 showing high mortality and absence of pigmentation (Figure 1a).

Specific growth velocity (µ) values slightly decreased in the cell cultures exposed to 1, 2.5, and 5 mM when compared to the control, while duplication time increased from 4.15 h in the control to an average of 5.13 h in the first three concentrations (1, 2.5, and 5 mM). In the diauxic growth cultures, µ values were smaller than the control in both growth phases (Figure 1b), whereas duplication times were higher. The second significant growth phase in diauxic cultures showed lower µ values than the first one, with the exception of the two highest concentrations tested (22.5 and 25 mM), in which cells took a longer time to start growing exponentially during the first hours (Figure 1b). Duplication times during the first growth phase were around 7.83 h in those culture media containing between 10 mM and 20 mM of H_2_O_2_, being even higher at 22.5 mM and 25 mM. During the second growth phase, cells took an average of 12.76 h to duplicate their population. At the mild concentrations (10–20 mM), during the first growth phase, cells were still capable of growing normally for around 4 h after the addition of H_2_O_2_ before they entered a first latency. After approximately 10 h, cells started growing exponentially again until reaching stationary phase. At the highest concentrations, the initial latency took place right after the addition of the oxidant, thus, possibly indicating difficulties during growth.

**Figure 2:**
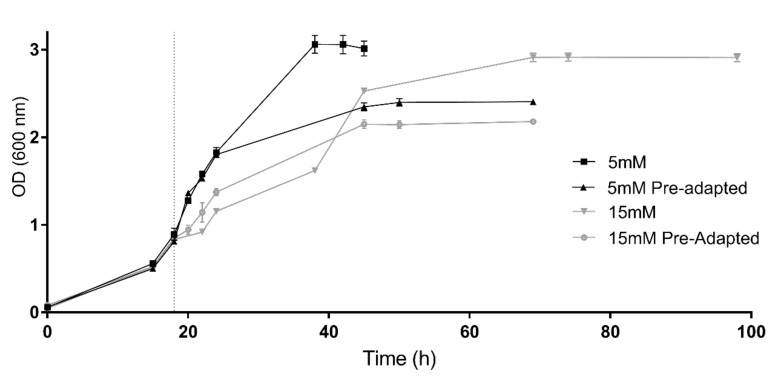
Growth of preadapted cells vs. growth of non-preadapted cells. Preadapted and non-preadapted *Hfx. Mediterranei* cell cultures were grown under 5 mM (black) and 15 mM (grey) H_2_O_2_ exposure. Vertical dotted line indicates time of the H_2_O_2_ addition.

Consequently, with the aim of evaluating the capability of adaptation, 5 mM and 15 mM conditions were tested again but using cells previously grown under these conditions as inoculum (preadapted cells) (Figure 2). At the beginning of the incubation, the cultures inoculated with 5 mM preadapted cells showed a similar growth tendency compared to those cultures containing 5 mM H_2_O_2_ inoculated with non-preadapted cells. However, cultures inoculated with preadapted cells reached an early stationary phase at an OD around 2.39. Despite this, an interesting behavior was observed in the preadapted 15 mM cell culture. Without previous adaptation, cells grown under this condition presented a diauxic growth curve; however, when these cells were re-inoculated in another culture with the same conditions, cells grew showing a normal exponential tendency and duplication time (6.47 h) was lower than the values observed in both growth phases of the non-preadapted culture (7.8 h and 10.9 h, respectively). Therefore, these results indicate an adaptation to the stressor. Nevertheless, it is worth mentioning that even if preadapted cells recovered the normal growth tendency, cells reached the stationary phase at a lower optical density value (OD 600 = 2.14) (Figure 2).

### 3.2. Carotenoid Production under Different H_2_O_2_ Exposure

#### 3.2.1. Variation in the Pigmentation under Different H_2_O_2_ Exposure

**Figure 3:**
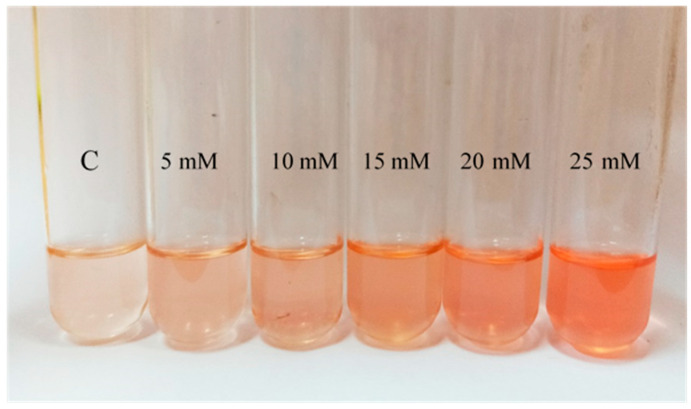
Visual comparison of the acetone extracts obtained from a selection of *Hfx. Mediterranei* cell cultures exposed to different H_2_O_2_ concentrations (0 mM ©), 5 mM, 10 mM, 15 mM, 20 mM, and 25 mM.

The general pattern observed in terms of pigmentation is an increase of the intensity of the color when cells are exposed to H_2_O_2_ (Figure 3). Comparing with the control, this variation was significant in those cultures containing between 1 mM and 25 mM of H_2_O_2_. In the presence of H_2_O_2_ concentrations above 25 mM, since growth was limited, there is a lack of pigmentation. However, although the differences in pigmentation are clear between control and cells exposed to the oxidant, it was impossible to conclude observationally from the cell cultures a concentration-dependent effect at the highest concentrations of H_2_O_2_ tested; however, this effect was much clear when looking at their acetone extracts (Figure 3).

#### 3.2.2. Bacterioruberin Concentration under Different H_2_O_2_ Exposures

**Figure 4:**
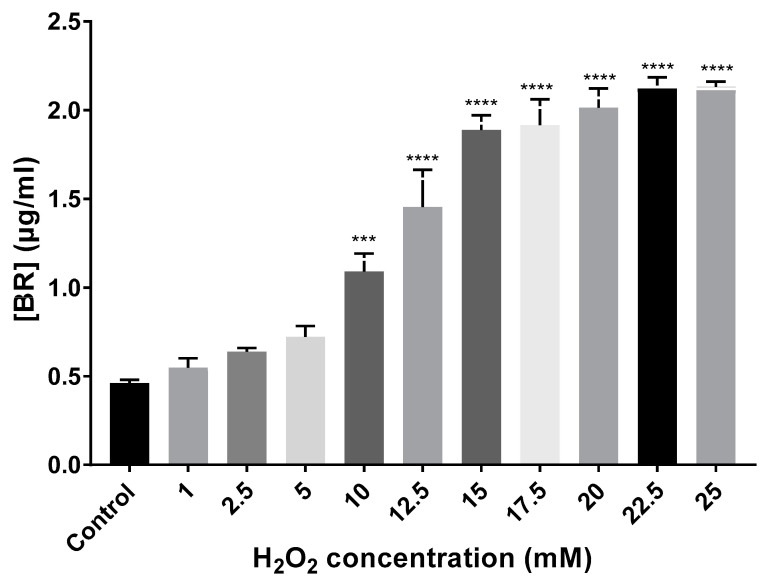
BR quantification (µg/mL) from extracts of *Hfx. Mediterranei* cell cultures exposed to different concentrations of H_2_O_2_. Each experimental sample was compared to the control to evaluate statistical significance. *** *p* < 0.001. **** *p* < 0.0001.

Total carotenoids were extracted, and BR content was quantified using the equation cited in Section 2.3 (Materials and Methods). BR production gradually increased in all cell cultures exposed to H_2_O_2_ compared to the control (no-treatment) until the growth limiting concentration of 25 mM. Control growing cells showed a BR production of 0.46 µg/mL, while the cell cultures exposed to H_2_O_2_ synthesized between 0.55 and 2.13 µg/mL. Thus, BR production showed a correlation with the increasing concentrations of the oxidant (Figure 4). Regarding BR concentration variation in the preadapted cell cultures, 5 mM preadapted and non-preadapted cells synthesized a similar concentration of BR (≅0.75 µg/mL), whereas preadapted 15 mM cells synthesized almost half of the concentration (1.09 µg/mL) observed when cells were not preadapted (1.89 µg/mL). 

### 3.3. Carotenoid Profile Obtained from Hfx. Mediterranei Acetone Extracts

**Figure 5:**
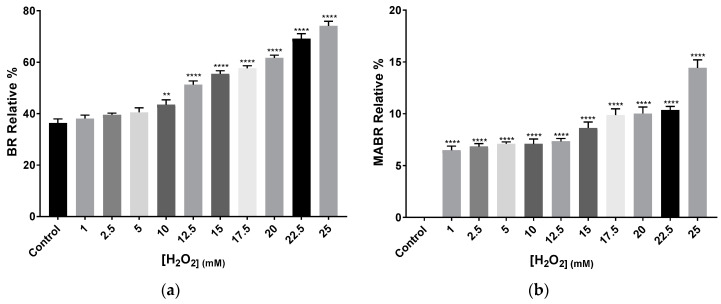
Relative percentage average of (**a**) BR and (**b**) MABR abundance and in *Hfx. mediterranei* cell cultures exposed to different concentrations of H_2_O_2_. Relative percentage was calculated by the sum of each percentage observed in the different peaks per sample in which BR and MABR were detected by its total m/z value: 740.9 and 722, respectively. Each experimental sample was compared to the control to evaluate statistical significance. ** *p* < 0.005, *** *p* < 0.001, **** *p* < 0.0001.

Acetone extracts obtained from cells grown in the presence of H_2_O_2_ were analysed by HPLC-MS. The analysis confirmed differences between the control and the cells exposed to H_2_O_2_, not only in terms of carotenoids abundance, but also in terms of carotenoids profile. Each of the 11 samples (including control) reported chromatograms showing between 12 and 24 peaks; each of them detecting several total or fragmented metabolites. Given the complexity of carotenoid identification from fragmented m/z values, this work focuses on the identified total m/z values clearly corresponding to a particular carotenoid based on the literature and previous works carried out by the authors. Regarding identified metabolites by their total m/z values, BR, and phytofluene were present in all samples in different abundances (Table 1; More details in Table A1, Appendix A). Since the m/z values of these compounds appeared in more than one peak per sample, a relative percentage was calculated. This relative percentage is calculated by the sum of each percentage of abundance of a particular m/z value found in two or more different peaks in one sample. In general, when comparing the control to H_2_O_2_ exposed samples, antheraxanthin, and epoxi-β-carotene were detected in the control, while they were absent in the rest of the samples. BR relative percentage increased with higher concentrations of the oxidant, reaching 73% at the highest concentration (Figure 5). The carotenoid precursor phytofluene was also very abundant in H_2_O_2_ exposed samples with relative percentages around 40–50%, whereas in the control it was less abundant (around 3.9% as relative percentage). This increase might indicate the activation of carotenoid synthesis pathways in response to oxidative stress.

Zeaxanthin was detected in all samples, either as total or fragmented m/z value. In those conditions where total m/z value was observed, it represented between 6% and 7% of the abundance of the peak. Lycopene, α-carotene, β-carotene, γ-carotene, and δ-carotene were also detected by their total or fragmented m/z values in all samples. However, given that all five share most m/z values, it is impossible to determine accurately the specific abundance of each of them per sample. Bisanhydrobacterioruberin (BABR) was not detected in any of the samples, whereas monoanhydrobacterioruberin (MABR) total m/z 722 was present in all of them with exception of the control. MABR relative abundance showed an increasing H_2_O_2_ concentration-dependent tendency, reaching a value close to the double observed in the initial concentrations, as in the case of BR relative abundance (Figure 5a,b)

## 4. Discussion

H_2_O_2_ is a natural by-product metabolite of the metabolism that at high concentrations can damage different cell structures due to oxidative stress mechanisms. Haloarchaea are known for synthesizing the rare carotenoid bacterioruberin and its derivatives as a protection mechanism against UV-induced oxidative stress [6] or exposition to low salt concentration [10,12,16]. However, there were no data until the date of this study about the effect of this natural oxidant on the production of BR in the case of extremophiles, particularly haloarchaea. Here, the effect of H_2_O_2_ on the carotenoid production of *Hfx. mediterranei* has been analyzed. *Hfx. mediterranei* cells were grown under normal conditions until an OD of 0.8 (mid-exponential phase). At this point, H_2_O_2_ was added and the effect on growth, total carotenoid production as well as BR production were observed until stationary phase. *Hfx. mediterranei* could grow successfully up to a concentration of 25 mM H_2_O_2_, which was much higher than the 3 mM tolerance observed in the case of *Hfx. volcanii* [26]. In the presence of higher concentrations than 25 mM, *Hfx. mediterranei* cultures reached an OD of approximately 1 at the stationary phase of growth and ultimately died (Figure 1). Specific growth velocity (µ) and duplication time (Dt) were calculated for each exponential phase of the cell cultures. Those cells exposed to higher concentrations than 10 mM displayed a characteristic diauxic growth that led to two different µ and Dt values. Regarding the culture containing lower concentrations of H_2_O_2_ (1, 2.5 and 5 mM), they showed a decrease in µ values and an increase in duplication times in comparison with the control. The same pattern was observed in the µ values of those with diauxic growth. The µ values of the first exponential phase were higher than those of the second one, with the exception of the two highest concentrations. Regarding Dt of 10–20 mM cultures, values around 7.8 h were observed in the first growth phase, while during the second growth phase, Dt reached values around 12.8h. Cells exposed to concentrations between 10 mM and 20 mM continued growing for a few hours after the addition of H_2_O_2_, while the exposure to higher concentrations led to an immediate latency. This behavior might be related to the toxicity of the compound since these concentrations are the limit of H_2_O_2_ that these cells can cope with. After several hours, cells started a new exponential phase until a stationary phase was reached. Given these results, it can be inferred that cells proceeded to a metabolic adaptation against the high concentrations of H_2_O_2_ and since they reached successfully the stationary phase, we can conclude that the machinery activated during this oxidative stress was outstanding overcoming the stressful environment. To confirm that cells adapted to these conditions, 5 mM and 15 mM cell cultures were repeated in this case being inoculated with preadapted cells to the mentioned concentrations. Previous works on microbial growth in the presence of toxic compounds revealed that cellular preadaptation to toxic or stressful conditions induce the activation of molecular mechanisms that may increase the tolerance of microbes to those toxic compounds [28,29]. In this study, a similar growth tendency was observed in the 5 mM condition when using preadapted cells as preinoculum. In the case of 15 mM, the diauxic growth observed with non-preadapted cells became a normal exponential growth curve, indicating that indeed cells adapted their metabolism to cope with high concentrations of H_2_O_2_. In both preadapted cases, stationary phase was reached at a lower absorbance than their non-preadapted controls; probably due to the fact the preadapted cells were already exposed to the stressor and the toxicity might have affected the ability of growth. In summary, previous exposure to the oxidant, although allowing adaptation, also hindered growth.

The presence of H_2_O_2_ in the cell culture media clearly influenced the pigmentation levels too. Observationally, a more intense orange-pinkish color can be noted in those cultures where cells were exposed to the oxidant. These results can support the idea of carotenoids as antioxidant mechanism previously described in the literature [6]. However, not only as a photoprotection mechanism as previously reported [30,31], but also against oxidant molecules such as the one tested in this study. Regarding the pre-adaptation experiment, BR concentration was diminished in the 15 mM preadapted cell culture. Hence, by comparing growth and pigmentation in the presence and absence of H_2_O_2_, it could be assumed that pre-adaptation reduced the stress caused to the cells, consequently leading to a reduction in the production of BR. Therefore, pre-adaptation at medium-high H_2_O_2_ concentration attenuated the stressful environment, turning diauxic growth into a normal exponential phase; however, since BR is synthesized as a stress response [12,16], the final concentration of the carotenoid was lower maybe due to lower oxidative stress status sensed by the cells [28].

Carotenoid extractions were carried out from each cell culture and BR production was quantified. All *Hfx. mediterranei* cell cultures exposed to H_2_O_2_ reported higher concentrations of the C_50_ carotenoids than the control. Even if these conditions were not the optimal for haloarchaeal carotenoid production [16], the highest concentrations of the oxidant led to levels of BR reported in the literature for the optimization of the synthesis of C_50_ carotenoids [32]. Therefore, the addition of an oxidant, such as H_2_O_2_, in combination with other environmental parameters, such as osmotic stress or temperature, could be useful to increase the synthesis of BR in biotechnology. However, higher concentrations than 25 mM led to a complete lack of pigmentation, which makes sense considering that growth was almost completely limited. It is worth highlighting that carotenoids are secondary metabolites synthesized during stationary phase [33] and given that in these mentioned cases, cells were not able to grow normally, lower levels or a total lack of carotenoids were expected. Considering that the oxidative stress caused by H_2_O_2_ at low-moderate concentrations (1–15 mM) improves the production of C_50_ carotenoids in *Hfx. mediterranei*, cell exposure to H_2_O_2_ could be part of an optimized protocol to produce BR at large scale for biotechnological purposes [12,13,16].

HPLC-MS analyses of acetone extracts from the cells grown in the presence of H_2_O_2_ at different concentrations reported between 12 and 24 peaks per sample, each of them detecting several total or fragmented m/z values. The high presence of fragmented values hampered the clear identification of carotenoids in this study. For this reason, we focused our study in those carotenoids identified by their total m/z values. BR and phytofluene were detected in all samples (control and all H_2_O_2_ concentrations tested) in different abundances. Given that the m/z values of these and other carotenoids were observed in more than one peak per sample, a relative percentage was calculated to determine the relative abundance of each metabolite. This relative percentage is calculated by the sum of each percentage of abundance of a particular m/z found in two or more different peaks in one sample. Antheraxanthin and 5,6-epoxi-β-carotene were only detected in the control, thus indicating that the synthesis of these compounds was stopped or at less reduced when cells were exposed to the oxidant. 5,6-epoxi-β-carotene is an oxidized variation of β-carotene, which could appear due to the exposure of the carotenoid extract to oxygen or to the activity of carotenoid cleavage oxygenases in the sample [34,35], since the functional diversification of carotenoids can be partly attributed to these enzymes. Interestingly, although the presence of antheraxanthin in the control and zeaxanthin in all H_2_O_2_ samples were detected, the genes involved in the synthesis of xanthophylls were not identified in a recent bioinformatic analysis [21], thus indicating that *Hfx. mediterranei* might have an alternative xanthophyll synthesis pathway, which would justify also de detection of other xanthophylls such as cantaxanthin in other species of the same genus [36,37]. BR and its derivatives were the major carotenoids. In fact, the relative percentage of abundance of BR clearly increased with the higher exposure to the stressor, confirming the results of the concentrations detected. The same concentration-dependent tendency is observed for MABR. The presence of these C_50_ carotenoids was in perfect agreement with the literature [38,39]; as a matter of fact, BR is the most abundant and always identified carotenoid in haloarchaea, although it has also been detected in some non-halophilic species such as *Rubrobacter radiotolerans* [40].Therefore, under H_2_O_2_ exposure, the production of BR and MABR is enhanced, while the synthesis of other carotenoids, such as zeaxanthin, appears to remain constant. Two different total MABR m/z values are reported in the literature: 722 [38,39,41] and 738 [16]. Most researchers seem to agree on using 722 to identify MABR, and in fact, in our samples, this value was detected in all samples with exception of the control and the behavior observed fits MABR. Nonetheless, 738 m/z value was detected in five samples out of 11; therefore, this value might be a different bacterioruberin derivative. Lycopene, α-carotene, β-carotene, γ-carotene, and δ-carotene all share most total and fragmented m/z values, and although their presence was observed in all samples, calculation of the relative abundance of each of them was impossible based on the approach here used. Since the synthesis of lycopene and β-carotene has been reported in haloarchaea [42,43] and the genes involved in their synthesis are identified in contrast to those of α-carotene, γ-carotene, and δ-carotene [21], these m/z values probably belong to lycopene and β-carotene. It is important to point out that this analysis detected several m/z values, which did not correspond to either total or fragmented m/z values from common carotenoids, and these metabolites were not clearly identified. Since most of these m/z values were ranging between 300 and 350, they could be alkanoates or derived metabolites with different methylation and/or oxidation states [44,45]. The presence of these unidentified compounds might be altering the usual published percentages of BR abundance [12,16,46]; however, despite this, BR is still the major identified carotenoid synthesised by *Hfx. mediterranei.* Phytofluene was also quite abundant under stress conditions, with relative percentages around 40–50%, while in the control they only appeared at a relative abundance of around 3.9%. The differences in the relative abundance of this carotenoid precursor suggest that oxidative stress induces the activation of carotenoid synthesis pathways, probably leading to an increase in BR synthesis, as this carotenoid is the most abundant among the identified metabolites and its concentration increases with higher oxidative stress conditions. This enhancement of carotenoid metabolism in response to H_2_O_2_-induced oxidative stress in *Hfx. mediterranei* coincides with results reported in yeast species such as *Neurospora crassa* [47], *Blakeslea trispora* [48], and *Phaffia rhodozyma* [49]. Opposite results have been reported in *Rhodotorula mucilaginosa* [50], in which carotenoid content is reduced in response to H_2_O_2_ exposure. In that case, this downregulation of carotenoid synthesis is argued by the pro-oxidant effect of an excess of β-carotene. Furthermore, in general the tolerance to the oxidant observed in yeast results is much lower that the one observed in *Hfx. mediterranei* [47,48,49,50].

## 5. Conclusions

In conclusion, this work demonstrates how *Hfx. mediterranei* cells modulates the synthesis of carotenoids in response to oxidative stress, leading to a gradual increase of the final concentration of bacterioruberin, the most abundant identified carotenoid. Therefore, oxidative stress by H_2_O_2_ induces modifications in the cytoplasmatic membrane, increasing the levels of BR to contribute to the cellular integrity either due to its role in the membrane or to its antioxidant activity or both. Be that as it may, further experiments are necessary to deepen in the understanding of this process. Additionally, *Hfx. mediterranei* can grow in the presence of H_2_O_2_ at concentrations up to 25 mM, which is one of the highest concentrations of this oxidant tolerated by an haloarchaea described at the time of writing this work. The stress caused by H_2_O_2_ promotes the synthesis of higher concentrations of C_50_ carotenoids in this haloarchaea, which could be of interest for those biotechnological processes aiming at the production of bacterioruberin at large-scale.

## Figures and Tables

**Table 1:** Most frequent identified carotenoids and carotenoid precursors in four representative samples (control and three concentrations within the range of H_2_O_2_ concentrations assayed in which cells were able to grow). The identification was based on their total m/z value in a selection of the tested conditions. Abundance is represented by the relative percentage calculated as the sum of each percentage observed in the different peaks per sample in which the carotenoid was detected by its total m/z value. More details are displayed in in Table A1, Appendix A.

Sample	Most Frequent Identified Carotenoids	
	Carotenoid (m/z (Total))	Relative Percentage (%)
Control	Bacterioruberin (740.9)	37.48
	5,6-Epoxi-β-carotene (553.7)	32.23
	Lycopene, α-carotene, β-carotene, γ-carotene, δ-carotene (537.6)	11.27
	Antheraxanthin (585.6)	5.25
	Phytofluene (542)	3.91
2.5 mM	Phytofluene (542)	49.71
	Bacterioruberin (740.9)	39.09
	Zeaxanthin (569.5)	7.79
	Monoanhydrobacterioruberin (722)	7.04
15 mM	Bacterioruberin (740.9)	54.62
	Phytofluene (542)	49.0
	Monoanhydrobacterioruberin (722)	9.04
	Lycopene, α-carotene, β-carotene, γ-carotene, δ-carotene (537.6)	5.99
20 mM	Bacterioruberin (740.9)	60.93
	Phytofluene (542)	32.97
	Monoanhydrobacterioruberin (722)	10.47

## Appendix A

**Table A1:** Most frequent identified carotenoids and carotenoid precursors by their total m/z value. Abundance is represented by the relative percentage calculated as the sum of each percentage observed in the different peaks per sample in which the carotenoid was detected by its total m/z value.

Sample	Most Frequent Carotenoids	
	Carotenoid (m/z (Total))	Relative Percentage (%)
Control	Bacterioruberin (740.9)	37.48
	Epoxi-β-carotene (553.7)	32.23
	Lycopene, α-carotene, β-carotene, γ-carotene, δ-carotene (537.6)	11.27
	Antheraxanthin (585.6)	5.25
	Phytofluene (542)	3.91
1 mM	Phytofluene (542)	42.47
	Bacterioruberin (740.9)	39.03
	Zeaxanthin (569.5)	6.75
	Monoanhydrobacterioruberin (722)	6.48
2.5 mM	Phytofluene (542)	49.71
	Bacterioruberin (740.9)	39.09
	Zeaxanthin (569.5)	7.79
	Monoanhydrobacterioruberin (722)	7.04
5 mM	Phytofluene (542)	47.16
	Bacterioruberin (740.9)	39.28
	Monoanhydrobacterioruberin (722)	6.98
10 mM	Phytofluene (542)	49.99
	Bacterioruberin (740.9)	42.13
	Zeaxanthin (569.5)	9.29
	Monoanhydrobacterioruberin (722)	6.76
12.5 mM	Phytofluene (542)	54.60
	Bacterioruberin (740.9)	50.27
	Monoanhydrobacterioruberin (722)	7.16
	Lycopene, α-carotene, β-carotene, γ-carotene, δ-carotene (537.6)	7.09
15 mM	Bacterioruberin (740.9)	54.62
	Phytofluene (542)	49.0
	Monoanhydrobacterioruberin (722)	9.04
	Lycopene, α-carotene, β-carotene, γ-carotene, δ-carotene (537.6)	5.99
17.5 mM	Bacterioruberin (740.9)	58.35
	Phytofluene (542)	47.55
	Monoanhydrobacterioruberin (722)	10.30
	Lycopene, α-carotene, β-carotene, γ-carotene, δ-carotene (537.6)	4.87
20 mM	Bacterioruberin (740.9)	60.93
	Phytofluene (542)	32.98
	Monoanhydrobacterioruberin (722)	10.47
22.5 mM	Bacterioruberin (740.9)	67.84
	Phytofluene (542)	14.04
	Monoanhydrobacterioruberin (722)	10.61
25 mM	Bacterioruberin (740.9)	72.93
	Phytofluene (542)	59.75
	Monoanhydrobacterioruberin (722)	13.89
	Zeaxanthin (569.5)	6.30
